# Patient and public understanding of antimicrobial resistance: a systematic review and meta-ethnography

**DOI:** 10.1093/jacamr/dlae117

**Published:** 2024-08-07

**Authors:** Gosha Colquhoun, Janyne Afseth, Ross Fagan, Fiona Thomson, Nicola Ring

**Affiliations:** School of Health and Social Care, Edinburgh Napier University, 9 Sighthill Court, Edinburgh, EH11 4BN, UK; School of Nursing, Midwifery and Paramedic Practice, Robert Gordon University, Aberdeen, Garthdee House Garthdee Road, Aberdeen, AB10 7QG, UK; School of Health and Social Care, Edinburgh Napier University, 9 Sighthill Court, Edinburgh, EH11 4BN, UK; School of Health and Social Care, Edinburgh Napier University, 9 Sighthill Court, Edinburgh, EH11 4BN, UK; School of Health and Social Care, Edinburgh Napier University, 9 Sighthill Court, Edinburgh, EH11 4BN, UK

## Abstract

**Objectives:**

To further develop an understanding of laypeople’s (adult patients and public) beliefs and attitudes toward antimicrobial resistance (AMR) by developing a conceptual model derived from identifying and synthesizing primary qualitative research.

**Methods:**

A systematic search of 12 electronic databases, including CINAHL, MEDLINE, PsycINFO, PubMed and Web of Science to identify qualitative primary studies exploring patient and public understanding of AMR published between 2012 and 2022. Included studies were quality appraised and synthesized using Noblit and Hare’s meta-ethnographic approach and reported using eMERGe guidance.

**Results:**

Thirteen papers reporting 12 qualitative studies were synthesized. Studies reported data from 466 participants aged 18–90 years. Five themes were identified from these original studies: the responsible patient; when words become meaningless; patient–prescriber relationship; past experience drives antibiotic use; and reframing public perception. These themes supported the development of a conceptual model that illustrates the tension between two different assumptions, that is, how can antibiotics be used for the collective good whilst balancing the immediate needs of individual patients.

**Conclusions:**

Findings suggest that AMR is a distinct ethical issue and should not be viewed purely as a prescribing problem. The meta-ethnography-generated conceptual model illustrates many factors affecting the public’s perception of AMR. These include laypeople’s own knowledge, beliefs and attitudes around antibiotic use, the relationship with the healthcare provider and the wider context, including the overwhelming influence of the media and public health campaigns. Future research is needed to explore effective health messaging strategies to increase laypeople’s baseline awareness of AMR as a public threat.

## Background

Antimicrobial resistance (AMR) is one of the top 10 global public health threats.^1^ The continuing emergence and spread of AMR poses a significant threat to public health and patient safety due to increasing numbers of infections becoming untreatable, and associated morbidity, mortality and healthcare expenditure.^2^ An estimated 5 million deaths worldwide were associated with bacterial AMR in 2019.^3^ This figure is predicted to rise to 10 million deaths annually by 2050, with a significant corresponding impact on the global economy of approximately 100 trillion US dollars, if no action is taken.^4^ The reasons for the increase in AMR are complex, but the emergence of resistant bacteria has been attributed to decades of excessive use of antibiotics.^5^ In high-income countries (HICs), the majority of antibiotics are prescribed in primary care settings.^6^ However, evidence shows that a substantial proportion of these prescriptions are unnecessary or inappropriate.^7,8^ This has prompted various initiatives to reduce inappropriate prescribing in primary care and coordinated efforts to understand levels of knowledge of antibiotic stewardship (AMS) amongst the general public.^9^

Antibiotic prescribing is influenced by a complex interplay between the knowledge, attitudes and behaviour of the prescriber and the patient.^10,11^ Previous studies have shown that patients overestimate the benefits of antibiotics, and their expectations can influence prescriber responsiveness to initiate a prescription.^12^ Numerous public health campaigns have been launched worldwide, including global events, such as World AMR Awareness Week (WAAW) to improve awareness among the public, prescribers and policymakers with the aim of engaging these groups to act.^13^ However, concerns remain about the lack of sufficient public-facing activities and evidence-based messaging during these events, with multi-country public awareness surveys indicating persistent public misunderstanding about the action of antibiotics against microbes and their prudent use.^14,15^

Internationally, there has been considerable research conducted to better understand the views of patients and the public of AMR but these studies have not been systematically reviewed. Our aim was therefore to systematically search for, identify and synthesize qualitative research from HICs, exploring patient and public attitudes and perceptions about AMR to build a conceptual model and help inform future AMS interventions.

## Methods

### Phase 1: Selecting meta-ethnography (ME) and getting started

Preliminary searching confirmed there was a suitably sized body of qualitative studies exploring patients’ and the public’s perceptions of AMR that could be synthesized using ME. Unlike other methods for synthesizing qualitative research, which amalgamate and describe primary study findings,^16^ ME aims to abstract findings and develop conceptual understanding to make ‘a whole into something more than the parts alone imply’.^17^ ME therefore suited our study aims. This inductive seven-step method is the most commonly utilized qualitative synthesis approach in healthcare research.^18,19^ The review protocol was registered on the PROSPERO International Prospective Register of Systematic Reviews (CRD42022324001). Ethical approval was not required.

### Phase 2: Deciding what is relevant

We sought primary research studies reporting on what adult patients and the public think and feel about AMR, their current understanding of the resistance issue and the language they use when discussing this topic (e.g. specific terms and phrases). A systematic literature search was conducted from January 2012 to December 2022. Conducting a search over the last 10 years was considered a pragmatic approach to ensure relevance to current antibiotic prescribing practices. Given that beliefs and social phenomena evolve, this period captures contemporary views and practices. Moreover, context is crucial for designing health interventions and assessing their applicability in various settings.^17,20^ There are significant differences between developed and developing countries in terms of healthcare infrastructure, access, resources and socioeconomic conditions.^21^ These differences, particularly in shared decision-making with patients regarding prescribing, and variations in organizational and professional risk thresholds, made it contextually more appropriate to focus on HICs.^11^ This approach aligns with the ME guidance, ensuring the findings are applicable to the context and setting of the planned intervention and strengthening the conceptual model.^17,22^ Non-English language papers were excluded due to resource constraints for translation and concerns about decontextualizing the findings and preserving the original meaning of quotes.^19,22^

We used the SPIDER tool (Table 1) to help with searching and locating relevant studies.^23^ We systematically searched 12 electronic databases: ASSIA, BASE, CINAHL, EMBASE, ERIC, MEDLINE via EBSCO, ProQuest Dissertation & Theses, PsycINFO, PubMed, Ovid Nursing, Web of Science and Google Scholar. To maximize return, we used extensive search terminology and relevant synonyms, including medical subject headings (MeSH). To enable transparent reporting in line with the eMERGe ME reporting guidance,^22^ a detailed search strategy is provided in Table S1 (available as Supplementary data at *JAC-AMR* Online).

**Table 1.** Search terms identified using the SPIDER tool^19^

**S**ample (patients or general public)	Patient* OR consumer* OR public* OR general public* OR population* OR people* OR communit* OR societ*
**P**henomenon of **I**nterest (understanding of antibiotic resistance)	Antibiotic* OR microbial* OR antimicrobial* OR drug* OR superbug* OR AMR OR resistanc* OR drug resistance OR anti-bacterial agent* AND understand* OR know* OR percept* OR perceiv* OR attitud* OR aware* OR belief* OR opinion* OR view* OR experience
**D**esign/**E**valuation/**R**esearch type (*qualitative)	Qualitative OR focus group* OR interview* OR ethnograph* OR observation*

*See Table S1 for hybrid qualitative filters.

**Figure 1.**
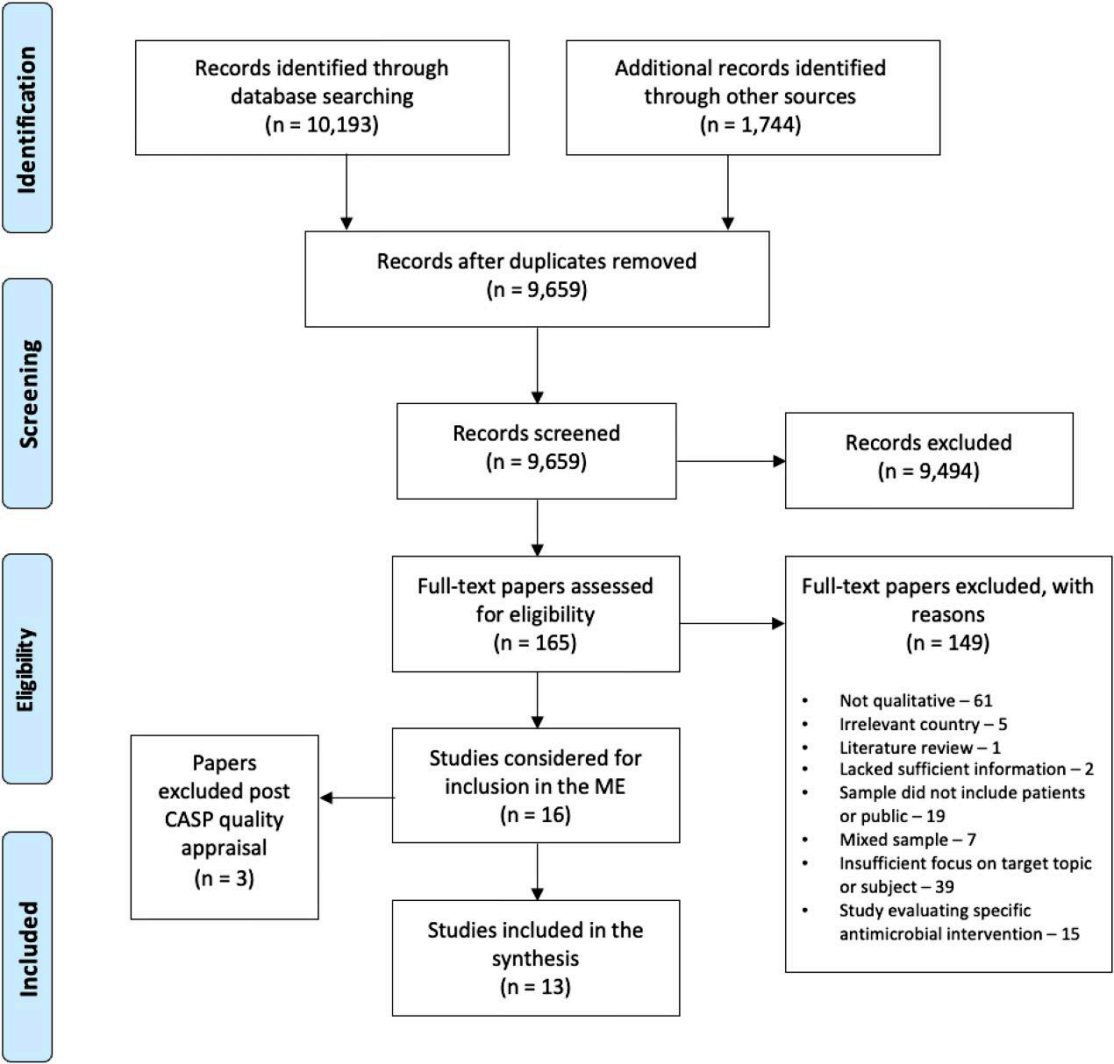
PRISMA diagram.^21^

We supplemented our online search with other methods, including hand-searching of relevant publications, reference screening and citation searching. Grey literature sources were searched for, including government reports, audits, conference proceedings and doctoral theses. Initially, employing a tailored search strategy developed by an academic librarian, potential studies for the ME were screened against our specific inclusion criteria (Table 2) using a rigorous simultaneous title and abstract screening process as recommended by the Institute of Medicine.^24^ Subsequently, full texts were assessed by two reviewers (R.F. and F.T.) working independently and then comparing outcomes. Any discrepancies were referred to the research team for arbitration. Literature searching outcomes were reported using PRISMA^25^ (Figure 1).

**Table 2.** Study inclusion and exclusion criteria

Inclusion criteria	Exclusion criteria
Primary research studies reporting adult patients’ and the general public’s views and understanding of antibiotics and AMR. Qualitative studies where there is clear identification, collection (e.g. interviews, focus groups) and inductive analysis (e.g. grounded theory, phenomenological analysis) of qualitative data. Mixed-methods studies only if the qualitative data are discrete and findings reported separately from quantitative findings. Studies carried out in HICs according to the international classification.^a^ Published in English language between 2012 and 2022.	Studies not reporting primary qualitative data collection and analyses (e.g. quantitative research, descriptive case studies, commentaries, editorials, reviews. Mixed-methods studies where qualitative data are not reported separately and cannot be extracted. Studies not containing direct quotations from research participants or where direct quotations cannot be obtained from a supplementary file or the study authors. Studies focusing on parent-related factors influencing antibiotic use in a paediatric population. Studies conducted in low- and middle-income countries. Studies where the full text is unavailable, or it is not written in the English language.

^a^See Supplementary data (Phase 2) for full definitions.

### Phase 3: Reading included studies

Studies meeting the inclusion criteria were read repeatedly and quality-assessed by two independent reviewers (R.F. and F.T.) using the Critical Appraisal Skills Programme assessment tool.^26^ Grey literature was appraised using the Authority, Accuracy, Coverage, Objectivity, Date and Significance (AACODS) checklist^27^ as recommended by NICE.^28^ As ME is interpretive, it was important to have conceptually rich texts.^18^ We therefore excluded ‘irrelevant’ papers and papers that lacked conceptual depth^29^ (see Supplementary data, Phase 3 for more details). This dual approach encouraged judgements on procedural aspects of research and helped us assess each study’s contribution to the final synthesis.^19,30^

Once we had agreed on which studies to include, we needed to familiarize ourselves with the key concepts in the data (key metaphors, phrases and meaningful ideas).^18^ First, to provide context for data interpretations, we entered study characteristics (e.g. participant demographics) into a template. Second, we reread all eligible studies multiple times to identify salient concepts in the introduction, results and discussion sections of each study. We then extracted original participant quotes (first-order) and authors’ interpretations (second-order data) verbatim into NVivo v12 qualitative software and then organized using a standardized data extraction form. To ensure rigorous analysis and minimize bias, the research team conducted regular discussions to verify and challenge the accuracy of interpretations. For example, two reviewers independently analysed the data and compared their findings, which were subsequently refined through team discussions.

### Phases 4 and 5: Determining how studies are related and translating studies

To determine the relationships between the studies and identify common and recurring meaningful ideas, we followed an approach recommended by Sattar *et al*.^31^ First, we created a list of the themes and concepts from each study and juxtaposed them against each other. From this list, we then clustered the concepts from different studies into relevant categories. This phase was iterative, and categories were revised through regular meetings. This facilitated translation, which was idiomatic and carried out chronologically, starting from the earliest publication. Translation of findings was reciprocal where similar concepts (albeit expressed differently) were drawn together and refutational, where contradictory or disconfirming concepts were noted. This process enabled us to ‘go beyond’ findings from individual studies, from simple descriptions of the data to developing higher interpretations. Translation led to the development of overarching themes.

### Phases 6 and 7: Synthesizing translations and expressing the synthesis

Through critical reflection and iterative discussion, Phase 4 and 5 findings were used to create a conceptual model. We did this by working from the identified themes to create a new line-of-argument (LOA), that is our ‘third-order (reviewer) constructs and interpretation’, a picture of the findings built on the individual parts of studies.^18^

## Results

We identified 11 937 possible references (Figure 1). Of those, 16 qualitative papers met our initial inclusion criteria. Following critical reading and quality appraisal (available in Tables S2 and S3), three papers were excluded: two were judged to lack conceptual and methodological depth,^32,33^ and one research report of a mixed-methods study^34^ was an exact replication of the Boiko *et al*.^35^ paper, in which the same research team reported qualitative findings separately. Thirteen papers reporting findings from 12 studies were finally included in the synthesis.^35–47^

The included studies reported the perspectives of 424 members of the public (health service users) and 42 hospital inpatients from various countries, including the UK,^35,40,46^ Sweden,^36,37^ Spain,^44^ Greece,^45^ multiple other European countries,^47^ Australia^38,42,43^ and New Zealand.^41^ All except one study^46^ provided gender information, and it is known that 168 (38%) participants were men. Study participants’ age ranged between 18^38,40,46^ and over 90 years.^35^ Only four studies provided details of participants’ ethnicity^35,40,41,44^ and eight indicated their education level.^36,37,40,41,43–46^ In many studies, the participants were highly educated, including those with a university degree^40,41,44,46^ and postgraduate education (including a doctoral degree).^36,37,43,45^

Sample size varied from 18^46^ to 99 participants.^42^ Data were collected using individual interviews (*n *= 6)^35,38,40,42–44^ and focus groups (*n *= 4).^36,37,39,45^ Three studies used interviews and focus groups.^41,46,47^ One study was a report rather than a peer-reviewed journal paper.^46^ Characteristics of the 13 papers, including publication year, country/setting, study focus, population, methods and findings, are detailed in Table 3.

**Table 3.** Summary of qualitative papers included in the synthesis (chronological order)

Study	Aim(s)	Sample	Data collection and analysis	Key findings
Wellcome Trust 2015^46^ UK	To get a deep understanding of how people think and feel about antibiotics, their current understanding of the resistance issue and the language they use around this area.	18 members of the general public Age range: 18–70+ Education: 6 university educated	Pair interviews and focus groups Data analysis not reported (grey literature publication)	4 overarching themes reported: people’s relationship with antibiotics; current knowledge and understanding of AMR and resistance; reactions to different ‘ways in’ of talking about resistance; current language and how it is understood.
Lum *et al.* 2017^43^ Australia	To investigate the perspectives, attitudes and behaviours of Australian consumers on antibiotic use and antibiotic resistance, and to inform national programmes for reducing inappropriate antibiotic consumption.	32 consumers Age range: 23–53 23 female/9 male Education: 32 with undergraduate/21 postgraduate degree	Semi-structured interviews Thematic analysis	3 main themes reported:prescription type; consumer attitudes, behaviours, skills and knowledge; consumer engagement with antibiotic resistance.
Ancillotti *et al*. 2018^37^ Sweden	To explore antibiotics-related beliefs and perceptions in Sweden.	23 members of the general public Age range: 20–81 13 female/ 10 male Education: 12 high school, vocational school and university diplomas; 8 had a Bachelor’s degree, vocational universities, and Master’s degree, 3 doctoral degree.	Focus groups Content analysis	3 overarching themes reported:perceived seriousness of, and susceptibility to, antibiotic resistance-related health issues; perceived benefits and barriers; self-efficacy in engaging in judicious behaviour and potential cues to engagement.
Zanichelli *et al.* 2019^46^ Belgium, Croatia, France, Netherlands, Switzerland	To explore inpatients’ experiences and views regarding antibiotics in five European hospitals.	42 hospital inpatients from 1 teaching hospital and 4 academic tertiary care centres Age range: 33–86 17 female/25 male	Interviews and focus groups Thematic analysis	6 themes reported: characteristics of the information received and missing information; patient preferences and expressed needs; sharing the information with family members: emotional support, alleviation of fear and physical comfort; perceptions and beliefs about healthcare workers, the ‘patient's role’ at the hospital and the decision-making process; bottlenecks in the organization of care at the hospital; perceptions and beliefs about AMR.
Boiko *et al*. 2020^35^ England	To investigate contemporary patient expectations and experiences of antibiotic prescribing in England.	31 patients who recently consulted GP for an infection Age range: 20–90+ 24 female/7 male Ethnicity: 25 white British, 3 white (other), 2 black, 1 Asian	Semi-structured interviews Thematic Analysis	5 themes reported: beliefs; expectations; experiences of taking antibiotics; experiences of antimicrobial resistance and side effects; experiences of consultations.
Davis *et al.* 2020^38^ Australia	To explore explanatory models for AMR and shed light on the persistence of the resistant body assumption and related concepts.	91 members of the general public Age range: 18–71+ 58 female/41 male	Semi-structured interviews Thematic analysis	5 overarching themes reported: evolution, ecology and climate, agriculture, mobility; hygiene; orthodox explanations; overuse and misuse; resistant bodies.
Essilini *et al.* 2020^39^ France	To explore the general public’s perceptions of antibiotic resistance, their attitudes around antibiotic use and expectations regarding awareness campaigns.	36 members of generic public 28 female/8 male	Focus groups Thematic analysis	3 overarching themes reported: knowledge and perceptions of antibiotic resistance; the ambiguous approach to antibiotic prescription; the social role of antibiotics.
Ghouri *et al.* 2020^40^ UK	To explore views about AMR in women who experienced urinary tract infections (UTIs) in pregnancy.	15 women who experienced UTI in pregnancy Age range: 18–43 Ethnicity: 14 white, 1 white (other) Education: 11 with university degree	Semi-structured interviews Thematic analysis	2 overarching themes reported: conceptualization of AMR; pregnancy as a deviation from the norm.
Lohm *et al.* 2020^42^ Australia	To explore the general public’s understanding of antibiotic use and AMR.	99 members of the general public 58 female/40 male/other Age and education not reported.	Semi-structured interviews Thematic analysis	3 main themes reported: deciding to seek antibiotics/waiting to determine the seriousness of the ailment before deciding to seek medical treatment; narrative on trust and expert knowledge of AMR and AMS; following the prescribed dosing.
Medina-Perucha *et al.* 2020^44^ Spain	To explore service users’ experiences of acute lower respiratory tract infection, the quality and access to healthcare services, and health education.	29 health service users Age range: 25–89 16 female/13 male Ethnicity: 28 white, 1 Latino Education: 1 postgraduate degree, 7 university degree; 6 high school; 9 primary school, 6 trade/certificate	Semi-structured interviews Content analysis	3 themes reported: risk perceptions and help seeking; treatment preferences and antibiotic use; relationship dynamics and communication with healthcare provider.
Ancillotti *et al.* 2021^36^ Sweden	To identify factors promoting and hindering a judicious approach to antibiotics.	23 members of the general public Age range: 20–81 13 female/ 10 male Education: 12 high school, vocational school and university diplomas; 8 Bachelor’s degree, vocational universities, and Master’s degree, 3 doctoral degree.	Focus groups Thematic analysis	3 main themes reported: justice; responsibility; demandingness.
Hika *et al*. 2022^41^ New Zealand	To explore the experiences, perceptions and beliefs that Māori have about antibiotic use in regard to acute upper respiratory tract symptoms, and of AMR.	30 members of the general public Age range: 20–77 23 females/7 males Ethnicity: Māori Education: incomplete information (3 university degree; 10 high school; 4 trade/certificate)	Semi-structured interviews and Focus groups Thematic analysis	3 overarching factors affecting antibiotic use reported: systemic; social; individual.
Papadimou *et al.* 2022^45^ Greece	To explore attitudes, perceived norms, and values in relation to antibiotics, and improve understanding of sociocultural determinants of antibiotic resistance in Greece.	20 members of the general public Age range: 21–55 12 female/8 male Education: 2 high school; 11 Bachelor’s degree, 7 Master’s degree.	Focus groups Thematic analysis	5 themes reported: norms; values; responsibility; scepticism; alternative practices.

Across all studies, 85 concepts emerged, which we organized into 11 higher conceptual categories (HCCs) that shared meaning (see Tables S4 and S5). From there, five overarching themes appeared: (1) the responsible patient; (2) when words become meaningless; (3) patient–prescriber relationship; (4) past experience drives antibiotic use; and (5) reframing public perception. Theme 1 arose from refutational analysis when it was noted that some translated findings described alternative dissonant perspectives of the same phenomenon. Themes 2–5 were derived from reciprocal translation (findings were compatible). Table 4 shows the studies supporting each theme. We present each theme with narrative exemplars below.

**Table 4.** Studies supporting each theme

Study	Theme				
	The responsible patient	When words become meaningless	Patient–prescriber relationship	Past experience drives antibiotic use	Reframing public perception
Wellcome Trust 2015^46^	✓	✓		✓	✓
Lum *et al.* 2017^43^	✓	✓	✓	✓	
Ancillotti *et al.* 2018^37^	✓	✓			✓
Zanichelli *et al.* 2019^46^	✓		✓		✓
Ancillotti *et al.* 2018^36^	✓	✓			✓
Boiko *et al.* 2020^35^			✓	✓	✓
Davis *et al.* 2020^38^	✓	✓	✓	✓	✓
Essilini *et al.* 2020^39^	✓		✓		✓
Ghouri *et al.* 2020^40^	✓	✓			✓
Lohm *et al.* 2020^42^	✓	✓	✓	✓	
Medina-Perucha *et al.* 2020^44^	✓		✓	✓	✓
Hika *et al.* 2022^41^			✓	✓	✓
Papadimou *et al.* 2022^45^	✓				✓

### Theme 1: The responsible patient

The essence of this theme is the tension between perceived individual health gains and society’s need to preserve antibiotic effectiveness. Although some participants voiced concerns that prioritizing collective health benefits may bring about undesired, and perhaps fatal, consequences from antibiotic treatments being withheld from vulnerable patients, across the studies there was strong support for prioritizing societal health benefits, for example:

*Yes, if we look at the big picture and think about how serious it’s starting to get…it’s a sacrifice you have to make, I think, to get a better situation. Society first. (Participant G4W2)* ^36^

However, participant differences were noted between different studies. For example, whilst participants in two European studies expressed a shared belief in there being personal responsibility for AMR, their perceptions of the collective dimension of responsibility diverged. For instance, participants in the Swedish study described the decreasing availability of effective antibiotics as a problem of justice^36,37^ but, this notion of collective responsibility was a vague concept for most participants in the Greek study.^45^ As illustrated below, a scenario in which society would share and act on the idea of a common purpose did not seem realistic for participants in this study:

*So our society does not have the [features] to deal with it. Beyond antibiotics, there is public confusion. We have lost track of things. (Participant G4M1*)^45^

In contrast, lay participants in other studies did not perceive AMR as a self-responsibility of the individual but as a phenomenon on which they could not act.^38–40^ Although concerned about AMR, they denied their own involvement in, and responsibility for, suboptimal antibiotic use.^39^ Some described that they felt overwhelmingly uncomfortable that the final decision whether to use antibiotics fell on them, rather than the GP.^43^ With the exception of one study, where participants were reluctant to transfer all responsibility to medical experts,^42^ there was also a strong sense of shifting the responsibility onto others. Although highly educated, most participants did not want such responsibility.

*Scientists out there will come up with something and they’re really clever, so I don’t worry too much because I think somebody’s solving the problem. (Participant CS23, female, 28 years old)* ^43^

While inpatients in Zanichelli *et al.*’s study^47^ doubted their ability to understand medical information, laypeople were unsure about how they could respond to AMR on an individual level and tended to assign the accountability for the AMR issue to healthcare professionals.^40^ Some participants believed that prescribing is solely an expert matter and therefore not under personal control.^38,48^ They rationalized the overuse and misuse of antibiotics by the willingness of clinicians to prescribe antibiotics too readily.

*… doctors are not really looking after their patients and just giving out antibiotics willy-nilly instead of giving it out when they’re really required. (Male, 60s, immunity illness)* ^38^

Knowledge about antibiotic consumption, resistance, and values such as altruism and trust in the healthcare system significantly influenced individual behaviour.^36,37,42^ In contrast to participants in the Essilini *et al*.^39^ and Papadimou *et al.*^45^ studies, other laypeople expressed high levels of self-efficacy to engage in judicious behaviours in relation to AMR.^37,40,42,44^

*The doctor decides*, *he tells me what to do*, *then I am responsible of what I do*, *if I do it well or I don’t do it*. *(Participant 23*, *male*, *69 years old)*^44^

Another area where participants’ views converged was when the needs of the individual outweighed any responsibility for AMR. In terms of life-threatening illness, the majority were critical of the idea of withholding antibiotic treatment in those situations to prioritize societal interest over individual needs. They emphasized the equal value of all human life.^37,45^ There was a sense that limiting individuals’ access to potentially beneficial treatment in the name of the greater good was morally questionable.

### Theme 2: When words become meaningless

This theme is underpinned by an understanding that apocalyptic narratives describing AMR are unhelpful and can be unsuitable when providing information about antibiotic resistance to the general public. Reflecting the proliferation of sensationalist language, participants spoke of ‘*disaster fatigue*’^37^ and ‘*scaremongering tactics*’,^46^ rendering AMR communication less effective. There was a sense that ‘antibiotic resistance’ is not a term that people instantly understand. Whilst some laypeople used the climate change analogy to describe AMR as ‘*a serious threat*’,^36,37^ others felt it was ineffective and as one participant described:

*Terms like superbugs and super-flu, they’re there to induce concern in the public. It’s a bit too much, we’re becoming desensitised to it. (Mixed-gender focus group, 18–25, at university, London)*^46^

Participants felt that future AMR disaster framing lacked personal relevance to them, and the vagueness of this threat caused uneasiness and uncertainty about when the disaster would become concrete.^36–38,40,46^ Some participants described that the dramatic language used in the media leads to people ‘*blanking out*’ the headline news stories about resistance.^46^ For example, participants in the Lohm *et al*. study^42^ highlighted how ambiguity of the scientific data presented to the public eroded compliance with medical advice and caused uncertainty. Some media messages were also described as confusing and undermined people’s confidence about their role in tackling AMR, such as:

*You get the odd media report saying that, you know, you shouldn’t finish the [antibiotic] course and your doctor’s telling you to finish the course, so I think there is a lot of misinformation about resistance. (Participant 3)*^40^

To better communicate risks associated with AMR, participants largely suggested using clear and simple language that is more personal to them and ‘*hits home*’.^38,40,46^ There was a sense across the papers too that laypeople take note only when the AMR threat feels direct and immediate. As one participant reported:

*It’s hard to conceptualise what is going to happen …, that just now [AMR] feels very abstract. I mean, what will happen is so far away… (Participant G2W1)*^36^

### Theme 3: Patient–prescriber relationship

This theme describes the desire to be heard and to be engaged in antibiotic prescribing decisions. When participants discussed the appropriateness of prescribing, they tended to refer to informed choice and shared decision-making. It was apparent that patients wanted information about their medical condition and treatment options without necessarily wanting the responsibility for making those decisions.^35,44^ Trust played a significant role in the decision-making process. Some participants reported they would accept the medical decision not to receive an antibiotic if they trusted the GP and the reason for this was clearly explained to them.^41,43,44^ For example:

*.. I also understand that there’s no point in treating some things with antibiotics … if that was clearly explained, I think I’d be less disappointed in the care that I receive from the doctor. (Participant CS05, female, 29 years old)*^43^

The importance of the relationship between clinician and patient was clear. GPs were described as more ‘*accessible*’ than hospital clinicians, and they were the preferred information source regarding the appropriate use of antibiotics.^39,43^ Participants perceived feeling distant from hospital doctors and that this negatively impacted on the quality of, and time for, their communication.^47^

*…there is a sort of fence, a barrier… When you are here, and they are above you, then it is more complicated, I find, to communicate with them. (Participant NL04, female, 61 years old)* ^47^

Some participants reflected on prescribers’ decision-making processes, and their own expectations for a prescription. If that expectation was not met, they felt frustrated.^44^ Some laypeople talked about how prescribers seek to meet patient expectations^35^ and described clinician willingness to prescribe antibiotics *‘willy nilly’*.^38^ Yet, most participants expressed an eagerness to avoid turning to antibiotics straightaway when unwell, perhaps a reflection of growing awareness of AMR risk:

*I’m wary of antibiotics. I would only use them in extreme circumstances because I do think they’re overused in our community. (Female, 71+ years old)* ^42^

Participants also described how they want to be listened to and taken seriously; to be given a thorough clinical examination and to have the GP explain the clinical findings, treatment options and decisions to them.^35,43^ However, the language used by doctors was not always understandable to many patients, and this underlined the need for patients to ask for information and clarification.^41,47^ In the Hika *et al*. study,^41^ participants (with lower educational status) described a hierarchy during the consultation with the doctor, where the doctor’s advice was seen as an important *‘direction*’ even if they did not understand the information about antibiotics. There was also a sense of vulnerability where participants described how they do not necessarily complain about such issues during consultations because of the doctor’s time constraints and their perceived role of the patient.^41,44,47^ Many believed that ‘*the doctor decides*’ and some expressed feelings of disempowerment or even loss of control, a barrier that prevented them from asking questions about their prescription(s).^44,47^

### Theme 4: Past experience drives antibiotic use

The studies revealed that an individual’s self-knowledge and past experience of illness are significant influencers of their perception of when they need antibiotic treatment.^35,38,42,44,46^ Some participants described how they used additional means to get their desired outcome of antibiotic prescription.

*I lied to him and said I’d had symptoms for 5–6 days and I hadn’t. I said I’m here for a prescription, I’m not moving. I was there about 10 minutes. (Mixed-gender focus group, 25–50, mixed education, London)*^46^

These antibiotic-seeking behaviours were strongly linked to previous experiences with similar infections and the treatment received. Past experiences of the speedy progression of the illness (with no side effects) prompted participants to seek antibiotics and avoid future episodes. Conversely, a negative experience (side effects and/or not working) decreased the likelihood of seeking or taking antibiotics.^42,43^ In one study, particularly women participants who had caring responsibilities, reported delaying antibiotic-seeking as they struggled to find time to access healthcare services.^44^ Whereas other participants depicted themselves as knowledgeable and reflexive patients or ‘*active agents*’ of their health treatment decisions,^42,44^ raising important questions of the ‘*right antibiotic being prescribed at the right time*’.^35^ In several studies, participants reported expecting to be prescribed an antibiotic when attending a GP consultation but this varied depending on the underlying cause of the infection and its impact on their daily life or their perception of being at risk—perhaps a reflection of growing awareness of AMR risk and knowledge of how antibiotics may affect their bodies.^35,42,44^

*I did expect that if it is something on the lungs, I would be given antibiotics. (Participant 18, male, chest infection)* ^35^

Participants’ accounts revealed that individuals with lower levels of formal education and/or less awareness of the topic held more misconceptions about antibiotic use and resistance. For instance, some were uncertain whether resistance pertains to the individual or the bacteria.^38,41,43,46^ These misconceptions were linked to non-compliance with medical advice in some studies.^42^ Furthermore, knowledge gaps regarding the nature of infections (viral or bacterial) and their appropriate management were also evident among certain participant groups.^38,41,46^

*No idea. It’s a bit beyond me all that stuff. (Male, 50s, no chronic illness)* ^38^

Participants further described how public expectations are more complex than previously reported. They reflected on some patients pressurizing prescribers, such as: ‘*I’m sure many people lie just to get antibiotics*’ (female, tonsillitis).^35^ There was also a strong sense of ‘validation’ connected to antibiotics as they were seen as ‘*proof of illness*’.^46^ For many, having a prescription meant a speedy recovery^44^ as antibiotics were considered a miracle treatment.^35,46^

*It’s like a magic pill. If I take these, I’ll be sorted (Mixed-gender focus group, 18–25, not university educated, Birmingham)* ^46^

Despite some participants understanding the ineffectiveness of antibiotics for viral infections, the non-prescription of antibiotics was seen as minimizing disease severity.^39^ Some participants described a sense of urgency and the need for a ‘*quick fix*’ for their infection.^44^ Reasons, such as being unable to take time off work/study, or fly (due to infection), being inconvenienced by a minor illness (e.g. an important event coming up) or persistence of worsening symptoms were reasonable grounds for expecting or requesting antibiotics.^42–44^ Participants often referred to antibiotics as something that would ‘*shift*’ their illness, but also as a symptomatic cure and something ‘*to boost the immune system with*’.^35^

### Theme 5: Reframing public perception

Participants identified the need for definitive long-term solutions despite the complexities the AMR issue presented, and they offered many suggestions for addressing misconceptions about antibiotics. In their discussions, they stressed the need for empowerment. They believed that receiving accurate information would enhance individuals’ perception of self-efficacy with regard to self-managing their health and thereby empower patients to engage in judicious antibiotic-use behaviour.^37,39,40,45^ Consistently clear and neutral messages about antibiotics from healthcare professionals, public health campaigns and media were seen as being productive in improving awareness of AMR.^35,37,39,41,43^ The clear need for better, more accessible health information materials that are tailored to an individual’s health literacy level was also apparent.^41,44,46^ As one person noted:

*Health literacy is important. I think that they [the public] need to know more, and health professionals need to know how to engage and educate you… so that they have an understanding of what antibiotics actually do. (Participant 28, female, 28 years old)*^41^

Participants suggested adopting clearer terminology, for example, referring to ‘antibiotic-resistant infections’ or ‘antibiotic-resistant bacteria’ rather than simply ‘antibiotic resistance’. There was also a need for key messaging to highlight the interdependence of individual action and societal consequences in AMR.^43^ Some also recognized the importance of highlighting the moral dimension of antibiotic use (preserving the efficacy of these drugs for future generations versus the patient’s own treatment expectations) within public and patient communications to foster judicious use.^36,40^ There was also a perception that patients seeking advice for common infections may benefit from better information concerning appropriate treatment options, including outlining risks to patients from prescribing and withholding of antibiotics.^35,36,46^

*People have to understand that they’re using [antibiotics] correctly when they really need to use them and that they don’t when it’s not necessary. (Participant G2M1)* ^36^

Many participants felt that the public should be better informed about AMR and be involved in the work to counteract this problem.^35,37,38^ Lack of awareness of what needs to be done to tackle AMR was perceived as a powerful barrier to individual patient change. Individual doctors were seen as having a role in raising awareness, but national patient education initiatives were also seen as being key to successful change. For example:

*So, doctors should … educate patients. It should be organised at the national level, some kind of programme of basic education for the patients, for example—not to take antibiotics for flu and viral infections. (Participant HR08, male, 40 years old)* ^47^

Most participants felt that finding more effective ways to provide information about antibiotics would not only raise awareness of AMR but also improve patients’ care experiences. For example, tailored strategies to inform hospitalized patients, and acknowledge their treatment concerns and preferences, may be useful to promote patient involvement and improve communication regarding antibiotic use.^47^

#### Line-of-argument

**Figure 2.**
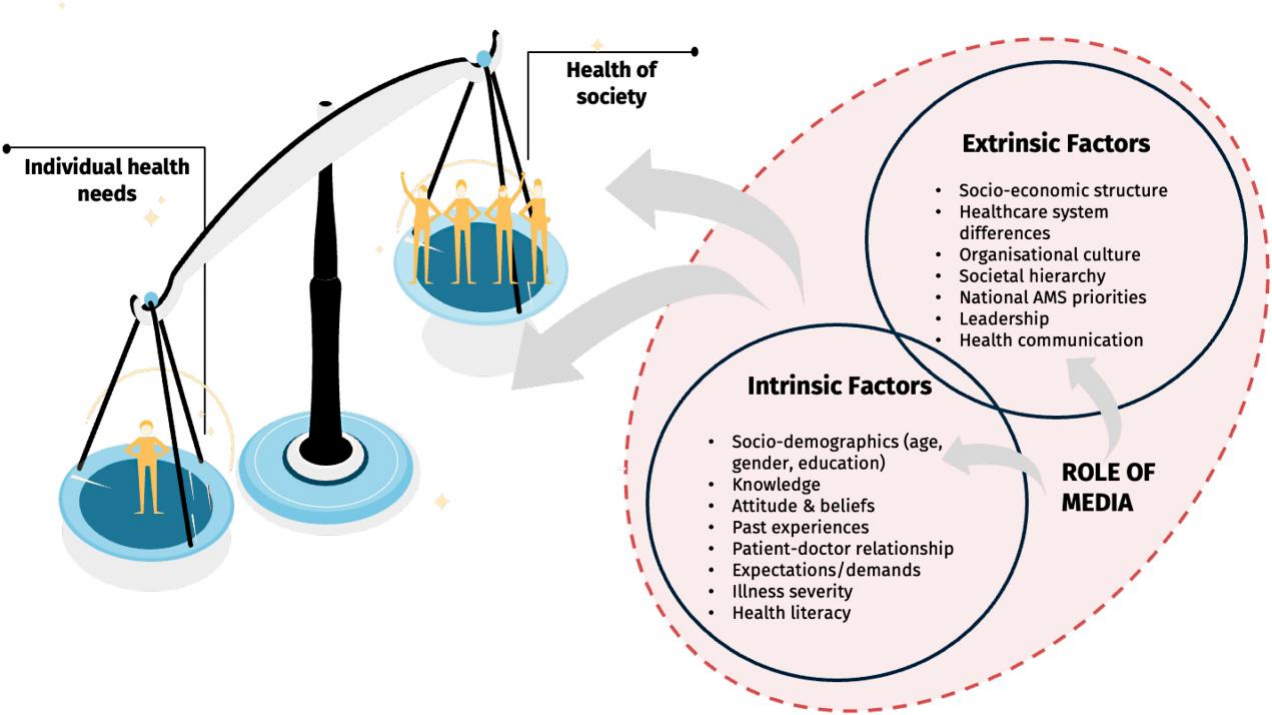
Balancing the health needs of individual and society—a conceptual model of patient and public understanding of AMR.

In keeping with the ME method, through team discussion and critical reflection on the included studies, themes were synthesized into an LOA. This reconceptualization of findings generated a new interpretation (Phase 6), enabling development of a conceptual model (Figure 2) of the patients’ and the public’s understanding and perspectives of AMR. This model illustrates that the laypeople’s ability to place societal needs before the individual depends on complex interplay of mutually dependent intrinsic and extrinsic factors. Among the former, knowledge, educational level, beliefs and attitudes around antibiotic use, and the relationship with the healthcare provider were the most cited factors influencing laypeople’s understanding of AMR, whilst the wider context, including the national structure and healthcare systems, were the most commonly reported extrinsic factors.

Through developing this model, which highlights the complexities around this topic, patient and public understanding of AMR can be summarized as hinging on negotiating the balance between two underlying assumptions about antibiotics (Figure 2). That is, how can antibiotics be used for the collective good against the health needs of individual patients. While the relationships between these assumptions will need to be tested in future research, at the core of the problem lies the social dilemma of two opposing standpoints (individual patient versus society), with mutually exclusive interests. This dilemma involves balancing the short-term interests of individuals with the long-term interests of current and future patients. This balance is influenced by unique contextual factors that vary across different regions, cultures, and countries, and is heavily shaped by the media’s role in influencing human attitudes and beliefs toward health and health behaviours. The extent of the media’s direct and indirect effects on health behaviours will, however, depend on various characteristics, including individual demographics and psychological traits.

## Discussion

In this novel ME, we synthesized findings from 13 qualitative studies focused on patient and public understanding of AMR. The themes identified enabled us to develop a conceptual model that adds depth and breadth to the existing knowledge base. While our findings have some overlap with concepts in the ethical debate literature,^48^ our analysis reveals important nuances related to moral sensitivity to the resistance issue that may warrant specific attention. The most striking findings were a distinct tension between individual and collective interests, and the perceived need for empowering the public through good health communications.

However, a dissonance between the two standpoints emerged from the analysis. On the one hand, the erosion of antibiotic effectiveness was perceived as a moral issue. Most participants seemed willing to place collective needs before the individual, but they were also concerned about personal risks. They were aware that the antibiotic resource is scarce, and that it would be unfair to consume it and leave limited to no antibiotic treatment options available to those who need them in the future. The use of antibiotics was perceived as morally acceptable when it is necessary for one’s care, but morally questionable in all other cases. Recent studies reinforce the notion that the public are significantly more willing to prioritize society over individual needs and show willingness to abstain from using antibiotics for the common good when they are made aware of AMR risks (i.e. when provided with knowledge of relevant facts).^49,50^ On the other hand, this moral responsibility for the collective good was in conflict with beliefs about the individual’s need for antibiotics to validate illness and accelerate recovery, and lack of concern about antibiotic harm and AMR. Previous research found that for many patients, being prescribed antibiotics has a great symbolic value.^51^

Specific notions on the effectiveness of antibiotics, expectations in terms of being given a prescription (even when not clinically needed) and compliance with regard to the medication all emerged as key factors associated with inappropriate antibiotic use. Yet, the analysis further shows that many people believe that they do not contribute to the development of AMR, and that the responsibility for tackling the resistance issue rests solely with others (e.g. clinicians, healthcare systems). This pressing need to recognize individual accountability for the emergence of drug resistance has been recognized internationally. While not a theme in our findings, this needs to be counterbalanced with factors driving the use of ‘over-the-counter’ and online purchases of antibiotics. These include inadequate regulatory enforcement mechanisms and the readiness of community pharmacists to dispense antibiotics without a prescription, which significantly contribute to self-medication behaviour among the general population, including in HICs.^52^ Moreover, the widespread practice of antibiotic sharing among the public globally—where individuals lend or borrow prescription medications intended for others—is a significant concern that warrants attention from healthcare providers.^53^ Efforts aimed at enhancing public awareness are also essential in addressing this issue.

Littmann and Viens^54^ argue that if the preservation of effective antimicrobials is in the interest of both current and future generations, then individuals globally, irrespective of regional or contextual differences, should be held accountable for the ignorant or unnecessary use of antibiotics or any other practices likely to accelerate the emergence of AMR. However, recent analysis of healthcare governance, encompassing processes, structures and organizational traditions, highlights ongoing challenges in coordinating efforts and managing antibiotic misuse in human health.^55^ Emerging research indicates that governance practices may significantly influence the development and spread of AMR, revealing notable disparities between and within countries.^56^ In the last decade, public perceptions have shifted from a position of full or assumed trust in healthcare professionals, to a more critical stance, necessitating increased transparency and accountability in both individual and organizational performance.^36,42,57^ Ethically, this poses challenging questions about how equity and fairness are to be incorporated and balanced with considerations of effectiveness, and whether it can be ethically justifiable to restrict antibiotic use to instances where their use prevents a substantial risk of irretrievable harm.^58,59^ Yet, as these drugs are a scarce global resource, it is crucial that they are used fairly—not only a moral, but also a practical necessity.^60^

Although there appears to be some improvement in public knowledge about AMR and appropriate use of antibiotics, and a decline in expectations for antibiotics, misperceptions about the problem held by participants at a conceptual level were particularly salient. For example, some participants did not know that antibiotics are ineffective against viruses and that resistance is not derived from the human body itself—even in countries where public awareness and education campaigns have been held.^61^ The idea that the body is itself becoming immune to the drug separates the individual from the society.^62^ The separate consideration of societal impact of resistance (manifested through public awareness of ‘superbugs’) gives rise to attribution of blame—whether to healthcare professionals, irresponsible patients or even the agricultural industry.^63^ Consequently, the public take the view that it is a problem for other people to resolve, with perceptions that it is other people who should reduce their antibiotic overconsumption. Research shows that the more distant the AMR consequences, the lower the perceived personal risks to patients from it.^64^ To think of AMR as some kind of future dilemma or discountable concern may in turn lessen individual responsibility for the problem and reduce the willingness to engage in a collective endeavour to preserve antimicrobials efficacy.

Similarly to our review, studies globally found that the general public agree that antibiotic overuse contributes to AMR, but far fewer understand their personal susceptibility and contribution to AMR.^65,66^ This lack of knowledge regarding the correct use of antibiotics and the desire for a ‘quick fix’, often exacerbated by the lack of patient-centred information provision, could lead to over-requesting these drugs.^67^ Literature shows that clinicians believe that most patients expect antibiotics,^68,69^ and that patients’ expectations to receive antibiotics are sufficient to actuate clinicians to prescribe them—even when they are not clinically justified.^70^ Yet, this ME highlights how patients’ expectations are now more complex than earlier research reported and exhibit tensions between adherence to antibiotics and consuming antibiotics in more reflexive, informed ways. This dichotomy reflects the wider discussion in the literature about direct patient demands for antibiotics, particularly in the context of increasing AMR, and doctors’ perceptions of such expectations.^71,72^ Power imbalance also came to the fore when participants perceived their clinicians to be experts. This complex dynamic highlights that patients can feel disempowered in relation to the medical experts who care for them, creating barriers for them to communicate their concerns and priorities.^73^ Given the recent increase in trust-based antibiotic campaigns (e.g. encouraging greater trust in the advice from healthcare professionals as to whether consumers need antibiotics or not),^13^ further research into the effect of trust on information provision in reducing inappropriate antibiotic expectations from primary care patients is required.

Educational attainment appeared to influence the ability to engage with the biological aspects of AMR, consistent with prior research suggesting that individuals from lower socioeconomic backgrounds may possess varying levels of awareness, knowledge and understanding of AMR compared with their counterparts from higher socioeconomic strata.^33,64^ Public surveys have shown that education from secondary school upwards is positively associated with greater knowledge about antibiotics.^74,75^ Yet, the literature shows conflicting results as to whether more knowledge about antibiotics is associated with more appropriate use.^76,77^ It needs to be acknowledged that people’s level of health knowledge and their ability to acquire and understand information differ, which could lead to misconceptions.^67^ Earlier research reported a discrepancy between what patients understand and what professionals think they understand, emphasizing the need for creating an environment where patients are heard, respected and valued as partners in their own care.^78^

We also found that the determinants of appropriate and inappropriate prescribing are not only situated in patient knowledge and behaviour, but also in the wider, sociocultural environment. A key finding was different (and often implicit) ideas about health, labelling of disease and coping strategies held by the public in different countries. These ideas shape both the expectations and the antibiotic-seeking behaviour of people in a country.^79^ Deschepper *et al*.^80^ relate the use of antibiotics in a country to a number of cultural characteristics of that country, as described in Hofstede’s model of cultural dimensions.^81^ For instance, Hofstede explains that power distance is concerned with how people holding different status communicate with each other. This was particularly relevant in participants’ accounts concerning moral sensitivity to the problem of AMR and patient–doctor relationships. A sense of personal moral duty to preserve antibiotic effectiveness and a preference for open discussion about the use of antibiotics was favoured in countries with low power distance and high levels of trust in clinicians (e.g. UK, Sweden and Australia), as opposed to hierarchical societies, such as France, Spain and Greece.

Central to this review was the finding that individuals consider themselves uncompliant with, or confused at times by, public health messages, not because they are irresponsible but because the environment in which they live sends contradictory messages about how best to tackle AMR. In terms of AMR communications, participants’ accounts did not correspond with official messages, indicating that much is yet to be done. The emergence of conflicting scientific advice, such as debate over when to cease the use of antibiotics staged in international news media, which is at odds with the ingrained public advice to ‘finish the course’,^57^ can lead to erosion of trust of medical expertise and pose challenges for global communications about AMR. News media perceived as conveying authority may also undermine AMR interventions because they promote messages of blame and social decline.^82^ This can be further exacerbated by the tendency of health communications to cast experts and laypeople in opposition.^83^ This situation has become more complicated since the COVID-19 pandemic. Although research has revealed a ‘rallying effect’ that boosted support for scientists and expertise during the pandemic,^84^ the sheer scale and depth of disruption caused by the pandemic meant that it was covered not just by health specialists, but by journalists and social media platforms, whose sourcing patterns and deviance from tenets of objectivity warrant attention.^85^ The significant increase in the endorsement of conspiracy theories has enabled and accentuated distrust toward health professionals and authorities, becoming a public health concern.^86^

Moreover, data suggest that the way the AMR problem is framed (e.g. ‘disaster’ or ‘apocalypse’) can influence perceived susceptibility negatively and hinder judicious behaviours in relation to antibiotic use, and this use of fear may increase avoidance of AMR messages.^87^ AMR resonates with the other stories of global crisis and catastrophe, such as climate change and the unprecedented destruction of ecosystems around the globe.^83^ Yet, from the viewpoint of patients and the public, some of the language used in the media fails to capture the complexity of AMR and the analogical reasoning may sometimes be inappropriate. Evidence from other health interventions shows that communications and media can reinforce blaming of the public as unknowing, ill-educated and resistant to expert advice.^88^

Numerous initiatives have been launched worldwide to appropriate antibiotic use, ranging from simple, low-cost internet campaigns to expensive mass-media efforts. A systematic review of 22 national and 6 regional campaigns revealed that multifaceted, sustained campaigns over several years yield the most significant effects.^10^ However, effectiveness was often hindered by the lack of a behavioural-change theory and uncertainty surrounding the key messages. Recent research indicates that public health campaigns that demonstrated improvement in their primary outcome measures typically utilized mass media for information dissemination, employed targeted messaging for specific infections, and emphasized interactions between healthcare providers and patients.^89^ As highlighted by Pinder *et al.*,^62^ AMR communication is inherently complex. The public faces conflicting messages: while urged to limit requests for antibiotics to combat AMR, they are also encouraged to seek early diagnosis and treatment for conditions such as cancer, heart disease and infectious diseases.

Effective communication strategies are urgently needed to reduce unnecessary antibiotic prescriptions. Yet, it remains uncertain which information provided by clinicians best reduces patient demand for antibiotics.^90^ Limited evidence exists on the effectiveness of specific terminology to discourage requests for antibiotics where they are not clinically indicated. Public health campaigns often focus on global AMR and societal harm, with little research comparing this with personalized messaging.^4,91^ The CDC and other stakeholders recommend shifting discussions to focus on individual harm, using simple understandable statements related to the patient.^92^ Clinicians and public health campaigns should emphasize the personal risks of non-indicated antibiotic use, such as potential irreversible changes to the human microbiome and the associated social and ethical implications (e.g. shared nature of the human microbiome across communities).^93^ Targeted interventions addressing this issue have often been overlooked, highlighting the need for greater awareness among medical professionals and clear guidelines from health policymakers.

Finally, the heterogeneity of culture, healthcare systems, consumption of antibiotics, and resistance to antibiotics across the globe most likely warrants different approaches for different countries.^10^ Earlier research has provided extensive evidence that different people exposed to the same message interpret it differently, depending on level of education, context and personal experience.^94^ How to engage with this complexity is an unresolved challenge for health media researchers across the world,^95^ and will undoubtedly require the generation of a new evidence base, with contributions from digital science and technology studies.^83^ Targeting limited resources to raise awareness among specific groups (e.g. GPs, hospital physicians, veterinarians, farmers and the general public) and employing behaviour change techniques tailored to each group’s current practices, motivations and individual context, supported by stakeholder involvement and follow-up, is one strategy to foster behaviour change.^96^ Co-creating person-centred and accessible educational and communication materials with the public, tailored to different age groups and/or learners’ cognitive abilities, could be another effective strategy.

## Strengths and limitations

Meta-ethnography is an interpretation of previously published data and, as such, this reflects the context(s) and experiences of the reviewers. As a multidisciplinary research team, with considerable experience in qualitative synthesis, we reviewed studies that incorporated views of 466 patients and the public across a wide range of ages, utilizing a range of qualitative methodologies and sociocultural contexts, and undertaken in a number of HICs, which adds to the transferability of our evidence synthesis. This is recognized as a strength of an interpretive paradigm that aims to reinterpret meaning across different qualitative studies and generate higher translations.^22^ The use of ME is well established, and we have previously utilized it to explore the antibiotic-prescribing behaviour in acute hospitals.^97^ However, we recognize the nuances of interpreting findings of primary studies and acknowledge that the original intended meaning from participants or original researchers may be lost in this process. We addressed this potential weakness by ensuring all stages of the review were checked for accuracy and were grounded in the data by constantly checking the findings against the original studies. We also reported our study using the eMERGe ME reporting guidance.^22^

A strength of this review is the comprehensive literature search strategy, including a large range of databases and grey literature, with robust quality appraisal of primary research. To inform the development of a novel AMR educational intervention for patients and the public, we focused on studies published in the last 10 years (2012–22). To enhance the quality of this completed ME, we updated our database searches in February 2024. One Norwegian study was identified that met our inclusion criteria.^67^ Its findings resonated with our themes and, if this study had been included in our synthesis, it would not have refuted our findings but provided equivalent translation. We also attempted to interpret the findings against the papers excluded during quality appraisal. This strategy ensured that important insights have not been missed, thus eliminating potential bias and adding to the credibility of the findings. For example, Davis *et al.*’s mixed-methods study,^32^ which included semi-structured interviews conducted with four patients in a US primary care setting (all female, with college-level education, aged 25–45 years old), raised an issue that was not captured in our review, relating to perceived inconsistencies in prescribing practices among clinicians, highlighting the challenges of effective health communication and its unintended consequences, such as the erosion of public trust. Whilst prescribing inconsistencies were not specifically reported in our analysis, including this paper would not have changed the outcome of our synthesis or the LOA as their key recommendations, such as trust and effective health communication, were included in our themes.

To ensure that the theory generated from synthesizing primary studies is relevant to the context, this review focused exclusively on studies published in English and conducted within the past decade in HICs. Including homogeneous studies strengthened the weight of the conceptual model; however, this approach may limit its transferability to non-English-speaking populations beyond these geographical areas. Further research is needed to assess the applicability of the developed model in low- and middle-income countries. Moreover, not all included studies reported details of participants’ characteristics, such as gender, ethnicity and level of education. This limitation restricted our ability to comprehensively identify differences in the public’s understanding and perceptions of AMR based on these demographics. It may also have limited our capacity to fully identify disconfirming cases across the studies. Additionally, our review specifically focused on adults and the majority of participants (62%) were women. Parent-related factors influencing antibiotic use in the paediatric population were beyond the scope of this investigation. This decision was made to ensure that the findings are directly relevant to the planned AMR intervention for adult patients. Further research exploring this populations’ perspectives of the resistance issue is underway.

### Conclusions

This ME provides a comprehensive review and discussion of the available qualitative evidence in relation to the general public and patients’ understanding of AMR in adult patients and the public. Through synthesizing findings, we demonstrated that unclear consequences of AMR remain abstract and problematic for the public to appreciate, and the scientific understanding of the factors that contribute to the resistance overall are either deficient or incorrect. The review reflects the significance of research interest to date, suggesting that effective communication plays a key role in improving the level of community awareness about healthcare issues. However, it is erroneous to assume that improving awareness will translate into positive change of behaviour, unless the issues are addressed holistically. Although the important question of how to tailor messages about AMR for specific population groups in different national settings remains unanswered, this review showed that messaging needs to be culturally relevant and adapted to the preferences of the target population. Findings emphasize the urgent need for understandable and accessible information regarding the science of AMR, its spread and prevention.

Finally, our findings suggest that existing public campaigns may not be effective, and renewed strategies that are multimodal, targeted and are informed by behaviour science are needed. A key consideration for AMR communications is that information provision can widen—not reduce—gaps between groups according to the level of education and access to media technologies. Therefore, AMR messages need to accommodate the need of diverse members of the public, including those who have fewer educational advantages. This calls for comprehensive research representing the voices of a more diverse public (including people with lower education status, of different ethnic backgrounds and more male participants) and a tailored communication strategy, which takes into account the various drivers of AMR and the solutions associated with it.

AMR is a global issue that calls for the collective effort of governments, the pharmaceutical industry, healthcare professionals and the general public to combat. The powerful cultural factor in explaining antibiotic use and the big differences between countries may provide useful direction for policymakers to intensify international cooperation in the area of antibiotic use and resistance.

## Supplementary Material

dlae117_Supplementary_Data

## Supplementary data

Tables S1–S5 are available as Supplementary data at *JAC-AMR* Online.
