# Supplementary material for: Patient and public understanding of antimicrobial resistance: a systematic review and meta-ethnography
Source: JAC Antimicrob Resist. 2024 Aug 7;6(4):dlae117. doi: 10.1093/jacamr/dlae117 (PMC11303694; doi:10.1093/jacamr/dlae117)
Supplement: dlae117_Supplementary_Data [file dlae117_supplementary_data.doc]

**Supplementary data: Details of applied methodology as informed by the eMERGe meta-ethnography reporting guidance.**

This review is based on Noblit and Hare's meta-ethnography (ME) approach**^1^** to synthesise qualitative research studies. Meta-ethnography consists of seven overlapping phases: getting started; deciding what studies are relevant; reading studies; determining how studies relate; translating studies into one another; synthesising translations; and expressing the synthesis**^1^**.

**PHASE 1: Getting started**

There are various methods for synthesising qualitative research, ranging from those aiming to describe or aggregate qualitative findings to those that are more interpretive and generate theory.**^2^** Meta-ethnography is a seven-phase, theory-generating, interpretive methodology for qualitative evidence synthesis (QES) developed by sociologists.**^1^** It is well suited to synthesising qualitative information to explore a phenomenon within a real-life context, such as antibiotic use.**^3^** We specifically chose this advanced method as it aims to generate novel interpretations that `go beyond` individual study findings to develop a new conceptual theory or a model whilst preserving the original meanings and contexts of the primary studies.**^4^** We used ME to develop novel insights and create a conceptual model pertaining to patient and public’s understanding of antimicrobial resistance (AMR) to inform the development of a robust behaviour change intervention for that population.

Our team included experienced interdisciplinary health professionals and social scientists (4 white females, including 3 European and 1 Canadian, and 1 European male) with an interest in antibiotic stewardship and expertise in qualitative evidence synthesis. GW, JA and NR had vast experience in conducting QES and NR was a member of the eMERGe team that developed the ME reporting guidance. We defined the key terms as follows:

- **`Antibiotic`** was defined as any type of therapeutic agent produced by an organism or made synthetically that selectively destroys or inhibits the growth of micro-organisms.**^5^** For simplicity, both terms `*antibiotics`* and `*antimicrobials`* were used interchangeably.
- **‘Antimicrobial resistance’** was defined as a natural phenomenon, which occurs when bacteria, viruses, fungi and parasites no longer respond to antimicrobial therapy. As a result of drug resistance, antibiotics and other antimicrobial medicines become ineffective and infections become difficult or impossible to treat, increasing the risk of disease spread, severe illness, disability and death.**^6^**
- **`High income countries`**: due to an array of disparities between developed and developing countries in terms of healthcare infrastructure, resources, access and provision as well as various social, cultural, political and economic conditions**^7^**, only studies carried out in countries with developed economies were considered for inclusion (e.g., UK, Europe, USA and Australia). Countries were classified according to the international classifications for the year 2022 by the Economic Analysis and Policy Division (EAPD) of the Department of Economic and Social Affairs of the United Nations Secretariat (UN DESA).**^8^** This approach ensured that the theory generated from synthesising primary studies reflects the function of the ME and is relevant to the context of the future intervention. Including relatively homogenous studies also helped strengthen the weight of the conceptual model.

A preliminary search confirmed that there was no QES developed *or* in progress that specifically addressed the topic of interest, and a sufficient number of primary studies existed that could be synthesised. This review formed the first stage in the development of a new behaviour-change intervention for the general public to improve their knowledge and understanding of antimicrobial resistance. Gaining in-depth and original insights from service users enabled us to develop a conceptual model which could inform practice and policy and guide further research in this field.

We registered our review protocol on the PROSPERO International Prospective Register of Systematic Reviews ([CRD42022324001](https://www.crd.york.ac.uk/prospero/display_record.php?RecordID=324001)).

**PHASE 2: Deciding what is relevant**

Details of the literature searching, screening and selection process are provided in the methods section of the paper and illustrated in the PRISMA diagram (Figure 1 within main manuscript).

Our search was informed by SPIDER (Sample, Phenomenon of interest, Design, Evaluation and Research type), designed to facilitate searching of qualitative and mixed-method studies.**^9^** With assistance from an academic librarian, we systematically searched 12 electronic databases and grey literature sources, including institutional repositories to search for dissertations and theses, conference proceedings and key organisations to search for reports and audits, such as the Wellcome Trust, Scottish Medicine Consortium, Department of Health & Social Care and National Institute for Health and Care Excellence (NICE).

Given the challenges of locating qualitative research, we applied a thorough and transparent methodological search strategy that could be replicated by others using the following:

1. **Electronic search strategy:**

The accessed databases included: ASSIA, BASE, CINAHL, EMBASE, ERIC, MEDLINE (via EBSCO), ProQuest Dissertation & Theses, PsycINFO, PubMed, OVID Nursing, Web of Science and Google Scholar. To maximise return, extensive search terminology and relevant synonyms were used, including medical subject headings (MeSH), supplemented by free-text and broad-based terms. The search strategy consisted of a combination of various search strings, including keywords such as: “antibiotic”, ‘antimicrobial resistance”, “public”, “patient OR consumer”, “understanding”, “experience” “attitudes” and “beliefs” (Table 1). The available evidence was then filtered through to identify qualitative studies using hybrid qualitative research filters originally developed by DeJean et al.**^10^** To ensure no studies were missed, the search was complemented by searching deep web sources (web pages that are not indexed and cannot be captured by performing standard searches using academic databases). The search was limited to a 10-year period (January 2012 and December 2022) to ensure that views and experiences reflected current policy and practice. We updated the search in February 2024 to check for any new publications.

**Table S1.** Example of search strategy applied in MEDLINE (EBSCO) including hybrid qualitative filters

| 1. (MH "Drug Resistance, Microbial") OR (MH "Drug Resistance, Bacterial+") 2. AB ( (antibiotic* or microbial* or antimicrobial* or drug* or superbug* or AMR) N2 resistanc* ) OR TI ( (antibiotic* or microbial* or antimicrobial* or drug* or superbug* or AMR) N2 resistanc*) 3. (MH "Drug Resistance") 4. (MH "Anti-Bacterial Agents+") 5. AB antibiotic* OR TI antibiotic* 6. S4 OR S5 7. S3 AND S6 8. S1 OR S2 OR S7 9. AB ( (patient* or consumer* or parent* or public* or general public* or population* or people* or communit* or societ*) N5 (understand* or know* or percept* or perceiv* or attitud* or aware* or belief* or opinion* or view* or experience* or thought*) ) OR TI ( (patient* or consumer* or parent* or public* or general public* or population* or people* or communit* or societ*) N5 (understand* or know* or percept* or perceiv* or attitud* or aware* or belief* or opinion* or view* or experience* or thought*) ) 10. (MH "Patients+") 11. (MH "Health Knowledge, Attitudes, Practice") 12. S10 AND S11 13. S9 OR S12 14. S8 AND S13 15. Qualitative Research/ 16. Interview/ 17. Nursing Methodology Research/ 18. (MM "Ethnology") 19. ethnograph$ 20. qualitative 21. ethnonursing 22. phenomenol$ 23. "life stor*" 24. (life stor*).mp. 25. theme* or thematic 26. social construct$ or (postmodern$ or post-struc-tural$) or (post structural$ or poststructural$) or post modern$ or post-modern$ or feminis$ or interpret$).mp. 27. (emic or etic or hermeneutic$ or heuristic$ or semiotic$).af. or (data adj1 saturat$).tw. or participant observ$.tw. 28. "action research" 29. (humanistic or existential or experiential or paradigm$).mp. 30. (field study or studies or research).tw. 31. human science 32. biographical method 33. theoretical sampl$ 34. ((purpos$ adj4 sampl$) or (focus adj group$)).af. 35. ((purpos$ adj4 sampl$) or (focus adj group$)) 36. ((purpos$ sampl$) or (focus group$)) 37. (account or accounts or unstructured or open-ended or open ended or text$ or narrative$).mp. 38. (life world or life-world or conversation analys?s or personal experience$ or theoretical saturation).mp 39. (lived or life adj experience$).mp 40. "cluster sampl*" 41. "observational method$" 42. "content analysis" 43. constant (comparative or comparison) 44. ((discourse$ or discurs$) analys?s) 45. "narrative analys?s" 46. TX ("semi-structured" or semistructured or unstructured or informal or "in-depth" or indepth or "face-to-face" or structured or guide) N3 (interview* or discussion* or questionnaire*) OR TX (focus group* or qualitative or ethnograph* or fieldwork or "field work" or "key informant" or phenomenograph*) 47. (MH "Interviews as Topic") 48. (MH "Focus Groups") 49. (MH "Narration") 50. S19 OR S20 OR S21 OR S22 OR S23 OR S24 OR S25 OR S26 OR S27 OR S28 OR S29 OR S30 OR S31 OR S32 OR S33 OR S34 OR S35 OR S36 OR S37 OR S38 OR S39 OR S40 OR S41 OR S42 OR S43 OR S44 OR S45 OR S46 OR S47 OR S48 OR S49 51. S14 AND S50 52. Limiters: Date of Publication: 20120101-20221231; English Language |
| --- |

**2**. **Non-electronic search strategy was guided by the eMERGe reporting guidance,^4^ the authors’ previous experience and expertise in conducting ME (NR, GW), and advice from the academic librarian. The strategy included:**

- **Reference checking** from key primary studies, studies included in systematic reviews and the studies included in this review.
- **Citation pearl searching** of the included studies using the `Cited by` option on Web of Science and Google Scholar, and the `Related articles` option on PubMed and Web of Science.
- **Hand searching of key journals**: issues of Antibiotics, BMJ Open, JAC Antimicrobial Resistance, British Journal of General Practice, Antimicrobial Resistance and Infection Control and PLOs One to ascertain the completeness of the search strategy.
- **Contact with experts:** leading authors in the field were contacted by email for comments and suggestions on key publications, also a list of items that could potentially be included in the review.

We exported the harvested records to EndNote bibliographic software and screened against eligibility criteria (see main manuscript) in two stages. After the removal of duplicates, all electronic records were initially screened for inclusion by title and abstract by two independent reviewers (RF and FT). Where title and abstract were equivocal, the full text paper was then read to make a definite decision on the relevance of the study for inclusion in the final synthesis. Both reviewers conducted full-text evaluation (*n*=165). Where consensus regarding inclusion could not be reached (*n*=9), a third reviewer (GW) was consulted. When information was unclear or missing from potentially relevant papers, GW emailed the authors and asked for additional information. Out of 14 authors contacted, only 3 responded (Kistler, Ancillotti and Wellcome Trust representative). Any disagreement regarding eligibility of papers was discussed with the full team. Subsequently, of the 11 authors who did not respond to our requests for more information, 8 of their papers were included. On completion of Phase 2, we identified 16 papers.

**PHASE 3: Reading included studies**

The 16 papers were then read in full multiple times and quality appraised using the Critical Appraisal Skills Programme (CASP) tool**^11^** by two team members (RF and FT; 100% dual check). CASP has been widely used to determine inclusion of studies into ME.**^12^** Grey literature was appraised using the Authority, Accuracy, Coverage, Objectivity, Date and Significance (AACODS) checklist**^13^**, as recommended by NICE**^14^** and included among that organisation’s checklists for evidence evaluation. To ensure that evidence which lacks methodological integrity (inadequate, incomplete or ambiguous methodological reporting with a score of less than 7) was judged accordingly and that we had conceptually rich texts – a key aspect of ME**^12^**, the strengths and weaknesses of the evidence that could potentially influence the ME results were discussed with the research team. Papers which were judged to be ‘irrelevant’ from the perspective of our study aims and papers with a score of more than 7 but judged to be purely descriptive and potentially lacking conceptual depth were also addressed for an overall opinion to the whole research team**^15^**. This dual approach encouraged judgements on procedural aspects of research and helped us assess each study’s contribution to the final synthesis.**^16,17^**

Following detailed discussion within the research team and reconciliation of the quality assessment with the third reviewer (GW), consensus was reached to exclude three papers. One mixed-method study**^18^** was a duplicate of Boiko et al’s paper**^19^**, which reported the qualitative findings separately. Two other studies were excluded as they did not reach methodological threshold (CASP score less than 7).**^20,21^** Quality appraisal helps reviewers become familiar with the content of primary studies, understand their context and assess each study’s potential contribution to the final synthesis. Quality appraisal allowed us to identity ‘key papers’ for synthesis, that is those with ‘*thick descriptions*` (conceptually *rich* rather than *descriptive* accounts and which included contextual detail)**^16^** and rigorous analysis and papers that reported only superficial insights. On this basis, we excluded Davis et al.**^20^** who reported data from four participants as a follow up to a large survey and McNulty et al.**^21^** as it was judged to lack an in-depth interpretation of the data, but categorised e.g., Ancillotti et al.**^22^** as a key paper because of its reported depth of insight and its ability to contribute substantively to analysis. Details of quality appraisal are provided in Tables 2-3 below. Thirteen papers reporting 12 primary studies were therefore included in the next meta-ethnography phases.

**Table S2. CASP quality appraisal**

| **Study** | **Decision to retain for Phases 4-6**  ✘/**✓** | **1.**  **Clear research aims** | | **2.**  **Qualitative methodology appropriate** | | **3.**  **Research design** | | **4.**  **Recruitment strategy** | | **5.**  **Data Collection** | | **6.**  **Reflexivity** | | **7.**  **Ethical Issues** | | **8.**  **Data Analysis** | | **9.**  **Findings** | | **10.**  **Research Value** | |
| --- | --- | --- | --- | --- | --- | --- | --- | --- | --- | --- | --- | --- | --- | --- | --- | --- | --- | --- | --- | --- | --- |
|  |  | **R1*** | **R2*** | **R1** | **R2** | **R1** | **R2** | **R1** | **R2** | **R1** | **R2** | **R1** | **R2** | **R1** | **R2** | **R1** | **R2** | **R1** | **R2** | **R1** | **R2** |
| **Ancillotti et al. (2018)** | **✓** – **SP** | Y | Y | Y | Y | Y | Y | Y | Y | Y | Y | N | N | P | U | P | Y | Y | Y | Y | Y |
| **Ancillotti et al. (2021)** | **✓** – **KP** | Y | Y | Y | Y | Y | Y | Y | Y | P | Y | N | N | P | Y | P | P | Y | Y | Y | Y |
| **Boiko et al. (2020)** | **✓** – **KP** | Y | Y | Y | Y | Y | Y | Y | Y | Y | Y | N | Y | P | Y | P | Y | Y | Y | Y | Y |
| **Davis et al. (2017)** | ✘ | Y | Y | P | Y | P | Y | P | Y | Y | Y | N | N | N | Y | P | N | Y | Y | Y | P |
| **Davis et al. (2020)** | **✓** – **SP** | Y | Y | Y | Y | P | Y | P | Y | P | Y | N | P | N | Y | Y | Y | Y | Y | Y | Y |
| **Essilini et al. (2020)** | **✓** – **SP** | Y | Y | Y | Y | Y | Y | Y | U | Y | U | N | N | Y | Y | N | N | Y | Y | Y | P |
| **Gulliford et al. (2021)** | ✘ | Excluded – a duplicate of the qualitative data reported in Boiko et al. (2020) above | | | | | | | | | | | | | | | | | | | |
| **Ghouri et al. (2020)** | **✓** – **KP** | Y | Y | Y | Y | Y | Y | Y | Y | Y | Y | N | U | Y | Y | Y | Y | Y | Y | Y | Y |
| **Hika et al. (2022)** | **✓** – **SP** | Y | Y | Y | Y | Y | Y | Y | Y | Y | Y | N | N | P | Y | Y | Y | Y | Y | Y | Y |
| **Lohm et al. (2020)** | **✓** – **SP** | Y | Y | Y | Y | Y | Y | P | Y | Y | Y | N | N | P | Y | Y | Y | Y | Y | Y | Y |
| **Lum et al. (2017)** | **✓** – **KP** | Y | Y | Y | Y | Y | Y | Y | Y | Y | Y | N | Y | P | Y | Y | Y | Y | Y | Y | Y |
| **McNulty et al. (2013)** | ✘ | Y | P | Y | U | Y | Y | Y | Y | Y | P | Y | N | P | Y | P | U | P | N | Y | P |
| **Medina-Perucha et al. (2020)** | **✓** – **SP** | Y | Y | Y | Y | Y | Y | Y | Y | Y | Y | Y | U | P | Y | Y | Y | Y | Y | Y | Y |
| **Papadimou et al. (2022)** | **✓** – **KP** | Y | Y | Y | Y | Y | Y | Y | P | Y | Y | Y | P | N | N | Y | Y | Y | Y | Y | Y |
| **Zanichelli et al. (2021)** | **✓** – **KP** | Y | Y | Y | Y | Y | Y | Y | Y | Y | Y | N | Y | P | Y | Y | Y | Y | Y | Y | Y |

**Table S3.** AACODS checklist for critical appraisal of grey literature

| **Study** | **Decision to retain for Phases 4-6**  ✘/**✓** | **1.**  **Authority** | | **2.**  **Accuracy** | | **3.**  **Coverage** | | **4.**  **Objectivity** | | | **5.**  **Date** | | **6.**  **Significance** | |
| --- | --- | --- | --- | --- | --- | --- | --- | --- | --- | --- | --- | --- | --- | --- |
|  |  | **R2** | **R1** | **R2** | **R1** | **R2** | **R1** | | **R1** | **R1** | **R2** | **R1** | **R2** | **R1** |
| **Wellcome Trust 2015** | **✓** – **KP** | Y | Y | Y | P | P | N | | Y | Y | Y | Y | Y | Y |

**Individual decisions 1-10**: **Y** – Yes, **N** – No, **P** – Partially, **U** – Unable to determine.

**Final decision for inclusion/exclusion in the synthesis:**

1. **KP** - a key paper that is conceptually rich and could potentially make an important contribution to the synthesis; to be included in the review.
2. **SF** - a satisfactory paper; to be included in the review.
3. **U** - unsure whether the paper should be included.
4. **IRR** - a paper that is irrelevant to the synthesis (i.e., not a qualitative study, or not addressing the review question); to be excluded from the review.

Once the studies for inclusion in the synthesis had been agreed on, two authors (GW, JA) began by repeatedly reading the included studies to familiarise themselves with the key concepts in the data (key metaphors, phrases and meaningful ideas), the raw data of ME.**^16^** First, to provide context for interpretations, we extracted study characteristics onto a template, which we previously used in another ME.**^3^** Characteristics of 13 included papers are presented in the main manuscript.

The extraction of raw data was then performed verbatim in chronological order. A PDF copy of each paper was imported to NVivo V.12 Software and organised separately according to the levels of data (participant quotes (first-order) and original author findings and interpretations of data (second-order). As papers were re-read, direct quotes (first-order constructs) and authors` interpretations (second-order constructs) were coded under separate Nodes. Setting up an additional Node for Original Studies allowed identification of where concepts came from during later phases. We also set up a Node for excluded studies, which we went back to upon completion of the synthesis to check whether important insights had not been missed. Data were then organised using a standardised data extraction form (see Table 4 for an example).

**Table S4**. Example of a data extraction table

|  |
| --- |

**PHASE 4: Determining how the studies are related**

We carried out this phase in several steps using the approach recommended by Sattar et al.**^23^** First, we compared the 13 papers by their characteristics, including the author, year of publication, country/setting, study focus, population, data collection and analytic approach (Table 3 in main manuscript).

We then related the studies by their findings. Two independent reviewers (GW, JA) extracted and coded data line-by-line using. Metaphors, themes and ideas were then compared to check for recurring concepts across studies (meaningful ideas that developed by comparing particular instances) along with contextualised details of each study.**^12^** These were then juxtaposed against each other to examine the relationships between the key concepts. As we progressed, an emphasis was put on grouping common concepts into relevant higher conceptual categories (Table 5). New ideas were allowed to emerge iteratively without *a priori* assumptions. The data within each category formed the basis of translation in the next stages. Continual reference to the original studies and conserving their unique language was key in this process.

Through the constant comparison method, we developed 85 concepts across the 13 papers. Reflective discussions within the team enabled us to revise, organise and further collapse these concepts into 11 higher conceptual categories (HCC) that shared meaning. For example, `understanding own body` and `self-care strategies` later became a more encompassing HCC of ‘knowledge and skills’. This process was time- and labour-intensive but helped to make sense of the data and aid clarity.

**Table S5. Reducing key concept from each study into relevant categories**

|  |
| --- |

**PHASE 5: Translating studies into one another**

Comparing concepts across 13 papers and regularly discussing the arising ideas seamlessly led into translation of studies into one another. During this phase, we compared each concept from each paper with all the other papers to check for the similarities and differences between the concepts. This was similar to the method of constant comparison.**^24^** We arranged all papers chronologically and compared and contrasted the key concepts from paper one with paper two, synthesised them and compared the outcome with paper three, and so on. The interpretations and explanations provided by the study authors were treated as data, and subsequently compared and translated across the papers to achieve a synthesis. To aid synthesis, two reviewers completed a translation table separately (Table 6), which was subsequently discussed within the research team. Then, our initial broad grouping of ideas was gradually refined by merging and collapsing conceptual categories into five themes. This process enabled us to `go beyond` findings from individual studies, from simple descriptions of the data to developing third-order interpretations.**^1,2^**

**Table S6.** Example of a translation table

| **Descriptor** (groups of similar concepts clustered together/broad thematic headings) | **First-order constructs** (the primary data reported in each studies/ participant quotes) | **Second-order constructs** (primary authors’ interpretations of the data –  metaphorical themes, concepts, meaningful ideas) |
| --- | --- | --- |
| **When words become meaningless** | ‘Terms like superbugs and superflu, they’re there to induce concern in the public. It’s a bit too much, we’re becoming desensitised to it.’ (M/F, 18-25, at university, London) | AMR means nothing to people – they can’t even guess what it stands for; AMR is difficult to grasp; The analogy to climate change is ineffectual (Wellcome Trust, 2015, UK) |
|  | ‘You get the odd media report saying that, you know, you shouldn't finish the course and your doctor's telling you to finish the course, so I think there is a lot of misinformation about resistance.’ (P3) | Conceptualization of AMR; Conflicting messages about which behaviours to adopt (Ghouri et al., 2020, UK) |
|  | ‘I think that it could be that it’s hard to conceptualise what is going to happen. Like you said that just now it feels very abstract. I mean, what will happen is so far away...’ (G2W1) | Climate change analogy to describe the intangibility of AMR (Ancillotti 2021, Sweden) |
|  | ‘What is normal for you, sometimes is expected to be known by the other as well.’ (NL03, M, 62 years old) | The language used by doctors was not understandable for the common patient; Operational blindness (Zanichelli et al., 2019, Belgium, Croatia, France, Netherlands, Switzerland) |
|  | ‘They do write in medical language and not everyone is medical and then you ask the pharmacy and they’re only reading off what the bottle says or on the paper.’ (P16, F, age unknown) | Lack pf information concerning antibiotics for patients; Health literacy is important (Hika et al., 2022, New Zealand) |

Most studies were similar in focus and allowed *reciprocal translation* (themes 2-5). However, as the studies were compared and translated into one another, some concepts emerged as disparate and stood in opposition to each other. The process of analysis revealed that some of the individually translated findings described alternative or opposing perspectives of the same phenomenon. For example, we observed that there were contradictory concepts related to the ideas about health, labelling of disease and coping strategies held by the public in different countries. This dissonance added a new dimension and a new *refutational* theme 1 – ‘the responsible patient’ was formed (see Findings in main manuscript).

**PHASE 6: Synthesising translations**

During synthesis of translations, the themes were brought together and matched against authors` interpretations and participants quotes of the respective primary studies. This phase is described as ‘making the whole into something more than the parts alone imply’.**^1^** Third-order analysis was carried out by reflecting on findings from Phase 5 against the study characterises and our interpretations using a translation table, and involved a degree of conceptual innovation. This enabled us to reconceptualise the findings and generate a higher order interpretation of the data. Our themes were brought together and matched against original author interpretations and participant quotes of the respective primary studies. As reflection is critical in ME, this was achieved through frequent team discussions.**^4^**

On reflection within the team and revisiting the original studies, we observed that the overarching themes overlapped and demonstrated a tension between two underlying assumptions: that antibiotics are a collective good and the individual need for antibiotic treatment. For example, we noticed that these two standpoints are influenced by many factors, including people’s own knowledge, beliefs and attitudes around antibiotic use, the relationship with the healthcare provider and the wider context, including overwhelming influence of the media and public health campaigns.

Finally, we created a conceptual model or visual representation of the *line-of-argument* (LOA) that was drawn from, `*but more than the sum of`*, the final themes**^1^** (see Figure 2).

**PHASE 7: Expressing the synthesis**

Findings of this review are presented as narrative, a new conceptual model, supporting tables and supplementary material.

The anticipated audience for this synthesis are public healthcare practitioners and managers, professional bodies, policymakers and those responsible for designing antimicrobial stewardship interventions who may value the practical implications of the findings and also researchers who may be interested in the methodology.

**Limitations:** see Discussion section of the paper. Although the expertise in synthesising qualitative research was vast among the team members, ME is an interpretative approach and the development of the conceptual model was inevitably driven by the research team`s backgrounds and based on their subjective interpretations. We acknowledge that a different team may have drawn different conclusions.

Due to practical issues (i.e. a large number of eligible studies to work through, also the time and resource-based constraints of the project), the emphasis was placed on the development of new interpretations and a LOA in a rigorous manner rather than producing an exhaustive summary of all studies. Therefore, a threshold to the studies methodological standards was applied. A different approach of judging the ‘weight of evidence’ of each paper and ensuring that only studies that provided the conceptual richness and `thick accounts` of patients and public`s lived experiences were included in the final synthesis may have been justified. However, considering that there is currently no gold standard on appraising qualitative studies, the concern was that including studies with poorly reported methods may produce findings lacking credibility.**^16^** We therefore decided that the reported methods had to meet a certain degree of methodological `soundness` before inclusion in the synthesis. The critical appraisal using the CASP tool was judged appropriate for that purpose.**^2,4^**

The exclusion of studies describing views and experiences of parents or where the emphasis was put on parent-related factors influencing antibiotic use in the paediatric population may be contested and a more inclusive approach exploring more diverse perceptions across different population groups may have been warranted. However, this strategy was chosen to ensure that evidence obtained in this way was suitable to the area of focus. We also made the decision to exclude low-income countries to ensure that the conceptual theory generated from synthesising primary studies reflects the function of ME and is relevant to the context and setting of the planned antibiotic intervention in the UK.

**Strengths:** The novelty of this meta-ethnography is the generation of a higher translation that helps to conceptualise patient and public’s understanding of antibiotic resistance. The number of included studies (n=13) encompassed the desired criteria, and provided a body of knowledge that allowed us to examine the phenomenon of interest and conduct a ME.**^16^**

Although the conceptual model cannot be claimed to be definitive and represent all laypeople, it offers a unique lens, through which the views and experiences of patients and the general public can be considered. The synthesis was carried out in a rigorous and systematic way including a large range of databases and grey literature with a continuous input from an academic librarian and the experienced research team, undoubtedly reinforcing the credibility of the findings. Three authors (GW, JA, NR) had a vast experience in conducting and synthesising qualitative research, whilst NR and GW had a special expertise in using ME. NR was also involved in developing the ME reporting guidance as part of the eMERGe project to increase the transparency and completeness of the reports.**^4^**

There is little published guidance on updating a meta-ethnography and there is no set time interval after which a meta-ethnography becomes out-of-date. Redoing a new overarching ME or `knocking down and rebuilding the house` could potentially change the findings of the original meta-ethnography.**^25^** To enhance the quality of the ME, we repeated database searches in February 2024 and found one study**^26^** that met our inclusion criteria. However, we believe that including the study in the final analysis would have not refuted our findings but resulted in equivalent meaning.

To increase credibility of the review and ensure that the breadth and scope of the data are captured in the synthesis, findings were reviewed and discussed within the research team through regular briefing sessions, providing opportunities to reflect on developed ideas and then refine and analyse interpretations using multiple theoretical perspectives. Although de-contextualisation of qualitative findings can be debated among methodologists, the quality of this review and rigour applied through all the stages means that it is possible to transfer this *`collective consciousness`* of the public’s perceptions of antimicrobial resistance beyond the contextual boundaries and apply the new conceptual model within the broader context of healthcare research that requires identification of both social and clinical dimension.**^27^**

A key methodological strength of our synthesis is that after creating the LOA, we reflected on our interpretation of the findings against the papers excluded following quality appraisal. This strategy ensured that important insights have not been missed, eliminating potential bias and adding to the credibility of the findings. For example, Davis et al. mixed-methods American study**^20^** raised an issue that was not captured in our review relating to perceived inconsistencies in prescribing practices among clinicians, highlighting the challenges of effective health communication and its unintended consequences, such as the erosion of public trust. Whilst prescribing inconsistencies was not specifically reported in our analysis, including this paper would not have changed the outcome of our synthesis or LOA as their key recommendations, such as trust and effective health communication, were included in our themes and the LOA.

Lastly, the uniqueness of this work lies in the translation process that employed a combination of reciprocal and refutation analysis, which facilitated conceptual innovation that went above and beyond those found in individual studies. The commitment to include refutational data in the synthesis - cases that are exceptions or outliers – helped to enhance the understanding through the development of a LOA. The refutational translation acted as a reminder not to seek similarity alone and to question why some concepts `fit` better than others.**^28^**

**References:**

1. Noblit GW, Hare RD. Meta-ethnography: *Synthesising qualitative studies*. California: Sage Publications Ltd; 1988.
2. France EF, Ring N, Thomas R *et al.* A methodological systematic review of what’s wrong with meta-ethnography reporting. *BMC Med Res Methodol* 2014; 14: 119.
3. Wojcik G, Ring N, McCulloch C *et al.* Understanding the complexities of antibiotic prescribing behaviour in acute hospitals: a systematic review and meta-ethnography. *Arch Pub Health* 2021; 79: 1–19.
4. France EF, Cunningham M, Ring N *et al*. Improving reporting of meta-ethnography: The eMERGe reporting guidance. *BMC Med Res Methodol* 2019; 19: 25.
5. Brunton LL, Chabner BA, Knollmann BC. *The Pharmacological Basic Of Therapeutics*. The McGraw-Hill Companies Inc; 2011.
6. World Health Organization. Antmicrobial Resistance. World Health Organization, 2023. <https://www.who.int/news-room/fact-sheets/detail/antimicrobial-resistance>
7. World Health Organization. Health in the post-2015 development agenda: need for a social determinants health approach. World Health Organization, 2016. <https://www.who.int/publications/m/item/health-in-the-post-2015-development-agenda-need-for-a-social-determinants-of-health-approach>
8. United Nations. World Economic Situation and Prospects Statistical Annex. United Nations, 2022. <https://www.un.org/development/desa/dpad/wp-content/uploads/sites/45/WESP2022_ANNEX.pdf>
9. Cooke A, Smith D, Booth A. Beyond PICO: The SPIDER Tool for Qualitative Evidence Synthesis. *Qual Health Res* 2012; 22: 1435–43.
10. DeJean D, Giacomini M, Simeonov D *et al.* Finding Qualitative Research Evidence for Health Technology Assessment. *Qual Health Res* 2016; 26: 1307–17.
11. Critical Appraisal Skills Programme. CASP Qualitative Checklist. CASP, 2023. <https://casp-uk.net/casp-tools-checklists/>
12. Toye F, Seers K, Allcock N *et al*. Meta-ethnography 25 years on: challenges and insights for synthesising a large number of qualitative studies. *BMC Med Res Methodol* 2014; 14: 80.
13. Tyndall J. The AACODS Checklist is Designed to Enable Evaluation and Critical Appraisal of Grey Literature: Flinders University, 2010. <http://dspace.flinders.edu.au/jspui/bitstream/2328/3326/4/AACODS_Checklist.pdf>.
14. National Institute for Health and Care Excellence. Interim Methods Guide for Developing Service Guidance. NICE, 2014. <https://www.nice.org.uk/process/pmg8/resources/interim-methods-guide-for-developing-service-guidance-2014-pdf-2007974164165>
15. Dixon-Woods M, Booth A, Sutton AJ. Synthesizing qualitative research: A review of published reports. *Qual Res* 2017; 7: 375–421.
16. Campbell R, Pound P, Morgan M *et al*. Evaluating meta-ethnography: systematic analysis and synthesis of qualitative research. *Health Technol Assess* 2011; 15: 1–164.
17. Higgins JPT, Thomas J, Chandler J et al. Cochrane Handbook for Systematic Reviews of Interventions version 6.4. Cochrane, 2023. [www.training.cochrane.org/handbook](http://www.training.cochrane.org/handbook)
18. Gulliford MC, Charlton J, Boiko O *et al.* Safety of reducing antibiotic prescribing in primary care: a mixed-methods study. *Health Serv Del Res* 2021; 9: 1–126.
19. Boiko O, Gulliford MC, Burgess C. Revisiting patient expectations and experiences of antibiotics in an era of antimicrobial resistance: Qualitative study. *Health Expec* 2020; 23: 1250.
20. Davis ME, Liu TL, Taylor YJ *et al.* Exploring Patient Awareness and Perceptions of the Appropriate Use of Antibiotics: A Mixed-Methods Study. *Antibiotics* 2017; 6.
21. McNulty CAM. Nichols T, French DP *et al.* Expectations for consultations and antibiotics for respiratory tract infection in primary care: the RTI clinical iceberg. *Br J Gen Pract* 2013; 63: e429–e436.
22. Ancillotti M, Eriksson S, Godskesen T *et al.* An Effort Worth Making: A Qualitative Study of How Swedes Respond to Antibiotic Resistance. *Pub Health Ethics* 2021; 14: 1–11.
23. Sattar R, Lawton R, Panagioti M et al. Meta-ethnography in healthcare research: a guide to using a meta-ethnographic approach for literature synthesis. *BMC Health Serv Res* 2021; 21.
24. Cahill M, Robinson K, Pettigrew J *et al.* Qualitative synthesis: a guide to conducting a meta-ethnography. *Br J Occup Ther* 2018; 81: 129–37.
25. France EF, Wells M, Lang H *et al*. Why, when and how to update a meta-ethnography qualitative synthesis. *Syst Rev* 2016; 5: 1–12.
26. Bergsholm YKR, Feiring M, Charnock C *et al*. Exploring patients' adherence to antibiotics by understanding their health knowledge and relational communication in encounters with pharmacists and physicians. *Explo*r *Res Clin Soc Pharm* 2023; 12: 1–10.
27. Friberg F, Dahlberg K, Petersson MN *et al.* Context and methodological decontextualization in nursing research with examples from phenomenography. *Scand J Caring Sci* 2000; 14: 37–43.
28. Pilkington H. Employing meta-ethnography in the analysis of qualitative data sets on youth activism: a new tool for transnational research projects? *Qual Res* 2018; 18: 108–30.
